# 6-Bromo-1-butylindoline-2,3-dione

**DOI:** 10.1107/S1600536808042098

**Published:** 2008-12-17

**Authors:** Lei Ji, Qi Fang, Jian-dong Fan

**Affiliations:** a School of Chemistry and Chemical Engineering, Shandong University, Jinan 250100, Shandong Province, People’s Republic of China; b State Key Laboratory of Crystalline Materials, Shandong University, Jinan 250100, Shandong Province, People’s Republic of China

## Abstract

There are two independent mol­ecules in the asymmetric unit of the title compound, C_12_H_12_BrNO_2_. The C—C bond lengths of the two carbonyl C atoms of the five-membered rings are distinctly longer than a normal C*sp* ^2^—C*sp* ^2^ single bond. One of the mol­ecules makes parallel self-coupled (inversion) dimers by π–π inter­actions with phen­yl–phenyl inter­planar distances of 3.403 (2) Å. The other mol­ecule also forms self-dimers at longer phen­yl–phenyl plane distances [3.649 (2) Å]. In the crystal, a C—H⋯O interaction is seen.

## Related literature

For synthesis and applications, see: Kopka *et al.* (2006 ▶); Pirrung *et al.* (2005 ▶); Zhou *et al.* (2006 ▶). For related crystal structures, see: Goldschmidt & Llewellyn (1950 ▶); Palenik *et al.* (1990 ▶).
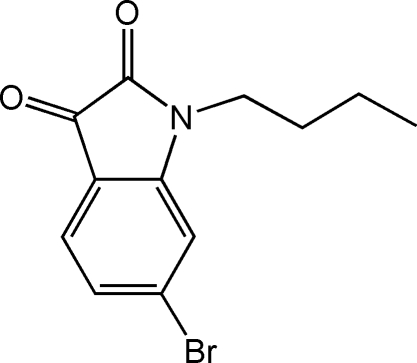


## Experimental

### 

#### Crystal data

C_12_H_12_BrNO_2_
*M* *_r_* = 282.14
Monoclinic, 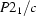

*a* = 13.3097 (2) Å
*b* = 11.8793 (2) Å
*c* = 16.2238 (2) Å
β = 112.340 (1)°
*V* = 2372.62 (6) Å^3^
*Z* = 8
Mo *K*α radiation
μ = 3.45 mm^−1^
*T* = 296 (2) K
0.37 × 0.13 × 0.11 mm

#### Data collection

Bruker APEXII CCD diffractometer
Absorption correction: multi-scan (*SADABS*; Bruker, 2005 ▶) *T*_min_ = 0.526, *T*_max_ = 0.744 (expected range = 0.487–0.689)
16713 measured reflections
5170 independent reflections
2424 reflections with *I* > 2σ(*I*)
*R*_int_ = 0.076

#### Refinement

*R*[*F* ^2^ > 2σ(*F* ^2^)] = 0.039
*wR*(*F* ^2^) = 0.070
*S* = 0.93
5170 reflections
364 parameters
1 restraint
H atoms treated by a mixture of independent and constrained refinement
Δρ_max_ = 0.39 e Å^−3^
Δρ_min_ = −0.49 e Å^−3^

### 

Data collection: *APEX2* (Bruker, 2005 ▶); cell refinement: *SAINT* (Bruker, 2005 ▶); data reduction: *SAINT*; program(s) used to solve structure: *SHELXL97* (Sheldrick, 2008 ▶); program(s) used to refine structure: *SHELXL97* (Sheldrick, 2008 ▶); molecular graphics: *SHELXTL* (Sheldrick, 2008 ▶); software used to prepare material for publication: *WinGX* (Farrugia, 1999 ▶).

## Supplementary Material

Crystal structure: contains datablocks I, global. DOI: 10.1107/S1600536808042098/cs2100sup1.cif

Structure factors: contains datablocks I. DOI: 10.1107/S1600536808042098/cs2100Isup2.hkl

Additional supplementary materials:  crystallographic information; 3D view; checkCIF report

## Figures and Tables

**Table 1:** Selected bond lengths (Å)

N1—C1	1.375 (3)
N1—C8	1.411 (4)
N1—C9	1.425 (4)
C1—C2	1.545 (4)
N2—C21	1.373 (3)
N2—C28	1.401 (3)
N2—C29	1.467 (4)
C21—C22	1.558 (4)

**Table 2:** Hydrogen-bond geometry (Å, °)

*D*—H⋯*A*	*D*—H	H⋯*A*	*D*⋯*A*	*D*—H⋯*A*
C7—H7⋯O3^i^	0.95 (3)	2.44 (3)	3.367 (5)	165 (2)

Symmetry code: (i) 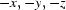.

**Fig. 1.**
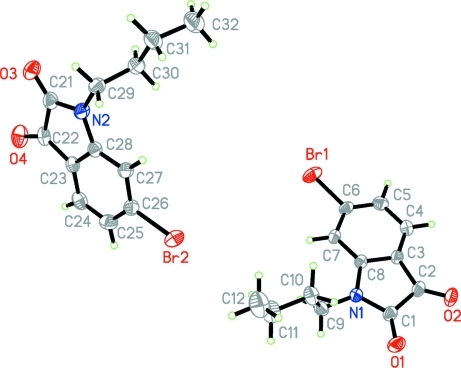
Two N-butyl-6-bromoisatin molecules in the asymmetric unit with 30% probability displacement ellipsoids

**Fig. 2.**
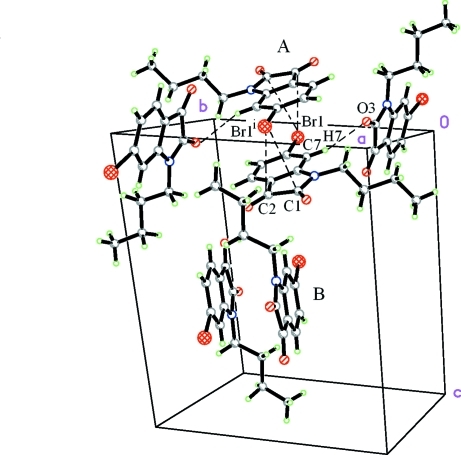
The molecular packing of the crystal showing the dimer structure [symmetry code: (i)-x+1, -y+1, -z]

## supplementary crystallographic information

### Comment

Isatin, 2,3-indolinedione is traditionally obtained from oxidation of oxindole and indigo blue. Its derivates have long been used as precursors of medicines and reductive dyes (Zhou *et al.*, 2006; Pirrung *et al.*, 2005; Kopka *et al.*, 2006). The first crystal structure report on isatin was by Goldschmidt and Llewellyn, 1950. Here we report the crystal structure of *N*-butyl-6-bromoisatin. There are two independent molecules in the asymmetric unit with very similar bond parameters. Except for the fused aromatic bond of C3≐C8 in one molecule and C23≐C28 bond in the other molecule, four other bonds in the five-membered heterocyclic rings can be classified as single C—C bonds and π-conjugated C≐N bonds. The C1—C2 (1.545 (4) Å) and C21—C22 (1.558 (4) Å) bond lengths are longer than expected for a C(*sp*^2^)-C(*sp*^2^) single bond. This may be the result of the repulsion of the lone pair electrons of the two oxygen atoms in *cis*-diones (Palenik *et al.*, 1990). Nitrogen atoms and their three bonded carbons are perfectly co-planar, showing that the *sp*^2^ N atoms allocate two p*_z_* electrons for π-bonding. The bond lengths of the π-conjugated N≐C bonds (c.f. Table 1) are much shorter than a single C—N bond length. Molecules are packed in dimers (Figure 2). One kind of the independent molecules are linked through a symmetry center to form dimer stack A while two other molecules form another dimer stack B. Mean planes of A and B dimers have a dihedral angle of 85.8 (1)°. The phenyl-phenyl spacing (3.40 (1) Å) in A dimers is considerably shorter than the spacing (3.65 (1) Å) in teh B dimers, indicating relatively stronger intermolecular π-π interactions between these A molecules. The intermolecular interactions in dimer A are further strengthened by C–Br short contacts. The C1···Br1[-x+1, -y+1, -z] and C2···Br1[-x+1, -y+1, -z] in dimer A are 3.476 (3) Å and 3.538 (3) Å, respectively. By comparison, the corresponding C···Br contacts in dimer B are much longer with the shortest distance being 3.672 (4) Å for C22···Br2[-x+1, -y+1, -z]. It may be the result of these different C···Br contacts that the C1—C2 bond length in dimer A is marginally shorter than the C21—C22 bond in dimer B. Amongst intermolecular C—H···O hydrogen bonds the strongest is the C7—H7···O3 one between dimers A and B (see Table 2).

### Experimental

6-Bromoisatin (10.5 g) was dissolved in 100 ml DMSO in a three-necked flask. Then KI (3.50 g), cetyltrimethyl ammonium bromide (1.00 g), and KOH (30.0 g) in 20.0 g water were added. Then 40.0 ml of n-bromobutane was added drop-wise into the above mixture with stirring. The mixture was stirred at 343 K for 2 days under nitrogen protection. The reaction mixture was washed by water, extracted with CHCl_3_, then the chloroform layer was dried by Na_2_SO_4_. After vaporizing the solvent, the crude product was purified by column chromatography, resulting in 9.5 g (yield 71%) *N*-butyl-6-bromoisatin product. The compound was dissolved in chloroform again. On most of the solvent evaporating at room temperature orange lump title crystals were formed.

### Refinement

All H atoms except those on the methyl groups were initially found in difference electron density syntheses and were used in the least-squares refinement. Six H atoms on two terminal methyls could also be located in the difference maps but some H—C bond parameters in the methyl groups became unreasonable. So HFIX 137 instructions were used to restrain the methyl H-positions.

### Crystal data

**Table:**

C_12_H_12_BrNO_2_	*F*(000) = 1136
*M**_r_* = 282.14	*D*_x_ = 1.580 Mg m^−^^3^
Monoclinic, *P*2_1_/*c*	Mo *K*α radiation, λ = 0.71073 Å
Hall symbol: -P 2ybc	Cell parameters from 3677 reflections
*a* = 13.3097 (2) Å	θ = 2.2–23.8°
*b* = 11.8793 (2) Å	µ = 3.45 mm^−^^1^
*c* = 16.2238 (2) Å	*T* = 296 K
β = 112.340 (1)°	Plank, orange
*V* = 2372.62 (6) Å^3^	0.37 × 0.13 × 0.11 mm
*Z* = 8	

### Data collection

**Table:**

Bruker APEXII CCD diffractometer	5170 independent reflections
Radiation source: fine-focus sealed tube	2424 reflections with *I* > 2σ(*I*)
graphite	*R*_int_ = 0.076
Detector resolution: 10.0 pixels mm^-1^	θ_max_ = 27.0°, θ_min_ = 1.7°
phi and ω scans	*h* = −16→17
Absorption correction: multi-scan (*SADABS*; Bruker, 2005)	*k* = −15→10
*T*_min_ = 0.526, *T*_max_ = 0.744	*l* = −20→20
16713 measured reflections	

### Refinement

**Table:**

Refinement on *F*^2^	Secondary atom site location: difference Fourier map
Least-squares matrix: full	Hydrogen site location: difference Fourier map
*R*[*F*^2^ > 2σ(*F*^2^)] = 0.039	H atoms treated by a mixture of independent and constrained refinement
*wR*(*F*^2^) = 0.070	*w* = 1/[σ^2^(*F*_o_^2^) + (0.0158*P*)^2^] where *P* = (*F*_o_^2^ + 2*F*_c_^2^)/3
*S* = 0.93	(Δ/σ)_max_ = 0.001
5170 reflections	Δρ_max_ = 0.39 e Å^−^^3^
364 parameters	Δρ_min_ = −0.49 e Å^−^^3^
1 restraint	Extinction correction: *SHELXL97* (Sheldrick, 2008), Fc^*^=kFc[1+0.001xFc^2^λ^3^/sin(2θ)]^-1/4^
Primary atom site location: structure-invariant direct methods	Extinction coefficient: 0.00147 (12)

### Fractional atomic coordinates and isotropic or equivalent isotropic displacement parameters (Å^2^)

**Table:**

	*x*	*y*	*z*	*U*_iso_*/*U*_eq_	
Br1	0.25474 (2)	0.52247 (4)	0.03130 (3)	0.07546 (17)	
O1	0.82928 (15)	0.3064 (2)	0.18511 (13)	0.0649 (7)	
O2	0.82426 (16)	0.5497 (2)	0.22161 (15)	0.0682 (7)	
N1	0.64300 (18)	0.3303 (3)	0.12570 (15)	0.0448 (7)	
C1	0.7490 (2)	0.3648 (3)	0.1675 (2)	0.0477 (9)	
C2	0.7445 (2)	0.4919 (3)	0.18633 (19)	0.0475 (9)	
C3	0.6286 (2)	0.5203 (3)	0.15321 (19)	0.0431 (9)	
C4	0.5754 (3)	0.6195 (4)	0.1517 (2)	0.0519 (10)	
H4	0.616 (2)	0.688 (3)	0.1810 (18)	0.058 (10)*	
C5	0.4628 (3)	0.6193 (4)	0.1150 (2)	0.0535 (10)	
H5	0.4240 (19)	0.690 (3)	0.1167 (16)	0.047 (9)*	
C6	0.4095 (2)	0.5211 (4)	0.0815 (2)	0.0476 (9)	
C7	0.4595 (2)	0.4196 (4)	0.0807 (2)	0.0435 (9)	
H7	0.4227 (18)	0.351 (2)	0.0576 (16)	0.036 (9)*	
C8	0.5720 (2)	0.4213 (3)	0.11826 (18)	0.0382 (8)	
C9	0.6102 (3)	0.2182 (3)	0.0965 (3)	0.0504 (10)	
H9A	0.538 (2)	0.218 (3)	0.040 (2)	0.073 (10)*	
H9B	0.659 (2)	0.185 (3)	0.081 (2)	0.078 (13)*	
C10	0.5921 (3)	0.1458 (4)	0.1651 (3)	0.0640 (11)	
H10B	0.537 (2)	0.185 (2)	0.1859 (17)	0.052 (9)*	
H10A	0.658 (2)	0.147 (3)	0.221 (2)	0.070 (11)*	
C11	0.5557 (4)	0.0275 (4)	0.1272 (3)	0.0831 (14)	
H11A	0.613 (3)	−0.012 (3)	0.121 (2)	0.100*	
H11B	0.477 (3)	0.032 (3)	0.085 (2)	0.100*	
C12	0.5496 (3)	−0.0541 (4)	0.1927 (3)	0.1061 (15)	
H12A	0.6201	−0.0628	0.2392	0.138 (10)*	
H12B	0.5253	−0.1253	0.1641	0.138 (10)*	
H12C	0.4993	−0.0276	0.2179	0.138 (10)*	
Br2	0.25658 (2)	−0.10901 (4)	0.09621 (3)	0.07726 (17)	
O3	−0.32733 (16)	−0.1957 (2)	0.03877 (14)	0.0728 (8)	
O4	−0.30063 (18)	−0.2190 (2)	−0.13304 (15)	0.0795 (8)	
N2	−0.14314 (18)	−0.1581 (2)	0.08915 (16)	0.0493 (7)	
C21	−0.2438 (3)	−0.1837 (3)	0.0266 (2)	0.0550 (10)	
C22	−0.2278 (3)	−0.1969 (3)	−0.0632 (2)	0.0538 (9)	
C23	−0.1117 (2)	−0.1769 (3)	−0.0404 (2)	0.0446 (8)	
C24	−0.0483 (3)	−0.1808 (3)	−0.0900 (3)	0.0552 (10)	
H24	−0.084 (2)	−0.193 (3)	−0.1480 (19)	0.060 (11)*	
C25	0.0612 (3)	−0.1618 (3)	−0.0495 (3)	0.0599 (10)	
H25	0.1067 (18)	−0.164 (2)	−0.0805 (16)	0.035 (8)*	
C26	0.1047 (2)	−0.1379 (3)	0.0399 (2)	0.0513 (9)	
C27	0.0439 (2)	−0.1332 (3)	0.0925 (2)	0.0501 (10)	
H27	0.0727 (17)	−0.120 (2)	0.1503 (16)	0.034 (9)*	
C28	−0.0653 (2)	−0.1544 (3)	0.0505 (2)	0.0443 (8)	
C29	−0.1191 (3)	−0.1497 (4)	0.1850 (2)	0.0545 (11)	
H29A	−0.1731 (19)	−0.188 (2)	0.1991 (16)	0.045 (9)*	
H29B	−0.056 (2)	−0.198 (3)	0.2144 (19)	0.068 (11)*	
C30	−0.1031 (3)	−0.0315 (4)	0.2208 (3)	0.0592 (11)	
H30B	−0.049 (2)	0.010 (3)	0.200 (2)	0.077 (11)*	
H30A	−0.162 (2)	0.016 (3)	0.195 (2)	0.067 (12)*	
C31	−0.0728 (4)	−0.0318 (4)	0.3219 (3)	0.0771 (13)	
H31B	−0.002 (3)	−0.071 (3)	0.355 (2)	0.112 (17)*	
H31A	−0.133 (3)	−0.073 (3)	0.333 (2)	0.103 (14)*	
C32	−0.0463 (3)	0.0819 (4)	0.3617 (2)	0.0941 (14)	
H32A	0.0122	0.1132	0.3483	0.129 (9)*	
H32B	−0.0249	0.0766	0.4252	0.129 (9)*	
H32C	−0.1090	0.1295	0.3376	0.129 (9)*	

### Atomic displacement parameters (Å^2^)

**Table:**

	*U*^11^	*U*^22^	*U*^33^	*U*^12^	*U*^13^	*U*^23^
Br1	0.03753 (19)	0.1118 (4)	0.0741 (3)	0.0122 (2)	0.01790 (17)	0.0168 (3)
O1	0.0405 (12)	0.082 (2)	0.0631 (16)	0.0147 (13)	0.0090 (11)	−0.0007 (14)
O2	0.0450 (13)	0.081 (2)	0.0662 (16)	−0.0181 (13)	0.0076 (12)	−0.0127 (15)
N1	0.0369 (14)	0.046 (2)	0.0473 (17)	−0.0027 (15)	0.0110 (12)	−0.0079 (17)
C1	0.0397 (19)	0.060 (3)	0.039 (2)	−0.0006 (19)	0.0107 (16)	0.001 (2)
C2	0.0372 (17)	0.062 (3)	0.038 (2)	−0.0066 (19)	0.0086 (15)	0.003 (2)
C3	0.0376 (17)	0.049 (3)	0.038 (2)	−0.0040 (19)	0.0091 (15)	−0.005 (2)
C4	0.056 (2)	0.045 (3)	0.051 (2)	−0.007 (2)	0.0165 (18)	−0.006 (2)
C5	0.053 (2)	0.051 (3)	0.056 (2)	0.010 (2)	0.0202 (19)	0.002 (2)
C6	0.0317 (16)	0.069 (3)	0.039 (2)	0.006 (2)	0.0095 (15)	0.005 (2)
C7	0.0361 (18)	0.049 (3)	0.042 (2)	−0.008 (2)	0.0114 (16)	−0.003 (2)
C8	0.0375 (17)	0.044 (2)	0.0305 (18)	0.0009 (18)	0.0100 (14)	0.0031 (18)
C9	0.052 (2)	0.043 (3)	0.053 (3)	0.005 (2)	0.016 (2)	−0.007 (2)
C10	0.072 (3)	0.050 (3)	0.064 (3)	−0.009 (2)	0.019 (2)	−0.004 (3)
C11	0.087 (3)	0.075 (4)	0.077 (3)	−0.005 (3)	0.020 (3)	0.003 (3)
C12	0.121 (4)	0.080 (4)	0.107 (4)	−0.018 (3)	0.032 (3)	−0.006 (4)
Br2	0.0453 (2)	0.0909 (4)	0.0969 (3)	0.0008 (2)	0.0285 (2)	−0.0097 (3)
O3	0.0427 (12)	0.090 (2)	0.0790 (17)	−0.0143 (13)	0.0160 (12)	−0.0119 (15)
O4	0.0672 (15)	0.094 (2)	0.0514 (16)	−0.0202 (15)	−0.0066 (13)	−0.0106 (16)
N2	0.0385 (14)	0.070 (2)	0.0342 (16)	−0.0056 (14)	0.0078 (12)	−0.0067 (16)
C21	0.048 (2)	0.059 (3)	0.049 (2)	−0.008 (2)	0.0089 (18)	−0.010 (2)
C22	0.055 (2)	0.042 (3)	0.052 (2)	−0.0093 (19)	0.0065 (18)	−0.004 (2)
C23	0.0489 (19)	0.043 (2)	0.036 (2)	−0.0037 (17)	0.0100 (17)	−0.0023 (19)
C24	0.072 (3)	0.050 (3)	0.036 (2)	−0.003 (2)	0.012 (2)	−0.003 (2)
C25	0.069 (3)	0.063 (3)	0.059 (3)	0.007 (2)	0.036 (2)	0.003 (2)
C26	0.0444 (18)	0.050 (3)	0.057 (2)	0.0006 (17)	0.0175 (18)	−0.001 (2)
C27	0.0423 (19)	0.064 (3)	0.038 (2)	0.0006 (18)	0.0095 (18)	−0.006 (2)
C28	0.0395 (17)	0.050 (2)	0.038 (2)	−0.0023 (16)	0.0089 (16)	−0.0028 (19)
C29	0.048 (2)	0.070 (3)	0.048 (2)	−0.007 (2)	0.0202 (19)	−0.004 (2)
C30	0.057 (2)	0.064 (3)	0.058 (3)	−0.006 (2)	0.023 (2)	−0.008 (3)
C31	0.103 (4)	0.076 (4)	0.065 (3)	−0.021 (3)	0.046 (3)	−0.020 (3)
C32	0.116 (3)	0.101 (4)	0.076 (3)	−0.027 (3)	0.048 (2)	−0.029 (3)

### Geometric parameters (Å, °)

**Table:**

Br1—C6	1.906 (3)	Br2—C26	1.907 (3)
O1—C1	1.214 (3)	O3—C21	1.209 (3)
O2—C2	1.210 (3)	O4—C22	1.208 (3)
N1—C1	1.375 (3)	N2—C21	1.373 (3)
N1—C8	1.411 (4)	N2—C28	1.401 (3)
N1—C9	1.425 (4)	N2—C29	1.467 (4)
C1—C2	1.545 (4)	C21—C22	1.558 (4)
C2—C3	1.468 (4)	C22—C23	1.465 (4)
C3—C4	1.370 (4)	C23—C24	1.370 (4)
C3—C8	1.395 (4)	C23—C28	1.391 (4)
C4—C5	1.387 (4)	C24—C25	1.371 (4)
C4—H4	0.99 (3)	C24—H24	0.89 (3)
C5—C6	1.366 (4)	C25—C26	1.372 (4)
C5—H5	1.00 (3)	C25—H25	0.92 (2)
C6—C7	1.379 (4)	C26—C27	1.382 (4)
C7—C8	1.386 (4)	C27—C28	1.374 (4)
C7—H7	0.95 (3)	C27—H27	0.88 (2)
C9—C10	1.496 (5)	C29—C30	1.503 (5)
C9—H9A	1.05 (3)	C29—H29A	0.95 (3)
C9—H9B	0.87 (3)	C29—H29B	0.98 (3)
C10—C11	1.536 (6)	C30—C31	1.532 (5)
C10—H10B	1.03 (3)	C30—H30B	1.04 (3)
C10—H10A	0.99 (3)	C30—H30A	0.93 (3)
C11—C12	1.464 (6)	C31—C32	1.481 (5)
C11—H11A	0.94 (4)	C31—H31B	1.00 (4)
C11—H11B	1.01 (3)	C31—H31A	1.01 (4)
C12—H12A	0.9600	C32—H32A	0.9600
C12—H12B	0.9600	C32—H32B	0.9600
C12—H12C	0.9600	C32—H32C	0.9600
			
C1—N1—C8	110.0 (3)	C21—N2—C28	110.9 (2)
C1—N1—C9	124.8 (3)	C21—N2—C29	123.9 (3)
C8—N1—C9	125.3 (3)	C28—N2—C29	124.9 (2)
O1—C1—N1	126.3 (3)	O3—C21—N2	127.2 (3)
O1—C1—C2	127.4 (3)	O3—C21—C22	127.0 (3)
N1—C1—C2	106.3 (3)	N2—C21—C22	105.7 (3)
O2—C2—C3	130.8 (4)	O4—C22—C23	131.7 (3)
O2—C2—C1	123.8 (3)	O4—C22—C21	123.5 (3)
C3—C2—C1	105.5 (3)	C23—C22—C21	104.7 (3)
C4—C3—C8	121.5 (3)	C24—C23—C28	120.3 (3)
C4—C3—C2	132.0 (3)	C24—C23—C22	132.2 (3)
C8—C3—C2	106.5 (3)	C28—C23—C22	107.4 (3)
C3—C4—C5	118.2 (4)	C23—C24—C25	119.6 (3)
C3—C4—H4	120.7 (16)	C23—C24—H24	115.6 (18)
C5—C4—H4	120.8 (16)	C25—C24—H24	124.7 (19)
C6—C5—C4	119.1 (4)	C24—C25—C26	119.0 (3)
C6—C5—H5	122.6 (15)	C24—C25—H25	122.3 (15)
C4—C5—H5	118.3 (15)	C26—C25—H25	118.7 (15)
C5—C6—C7	124.8 (3)	C25—C26—C27	123.4 (3)
C5—C6—Br1	118.4 (3)	C25—C26—Br2	119.2 (3)
C7—C6—Br1	116.8 (3)	C27—C26—Br2	117.4 (3)
C6—C7—C8	115.3 (3)	C28—C27—C26	116.3 (3)
C6—C7—H7	125.2 (15)	C28—C27—H27	120.7 (16)
C8—C7—H7	119.5 (15)	C26—C27—H27	123.0 (16)
C7—C8—C3	121.1 (3)	C27—C28—C23	121.4 (3)
C7—C8—N1	127.1 (3)	C27—C28—N2	127.3 (3)
C3—C8—N1	111.8 (3)	C23—C28—N2	111.3 (2)
N1—C9—C10	113.9 (3)	N2—C29—C30	114.5 (3)
N1—C9—H9A	110.8 (17)	N2—C29—H29A	110.0 (15)
C10—C9—H9A	107.0 (17)	C30—C29—H29A	111.5 (17)
N1—C9—H9B	111 (2)	N2—C29—H29B	106.1 (17)
C10—C9—H9B	108 (2)	C30—C29—H29B	112.8 (18)
H9A—C9—H9B	106 (3)	H29A—C29—H29B	101 (2)
C9—C10—C11	110.1 (4)	C29—C30—C31	110.6 (4)
C9—C10—H10B	108.8 (16)	C29—C30—H30B	109.3 (18)
C11—C10—H10B	112.8 (16)	C31—C30—H30B	115.0 (17)
C9—C10—H10A	109.7 (18)	C29—C30—H30A	115 (2)
C11—C10—H10A	114.1 (19)	C31—C30—H30A	109 (2)
H10B—C10—H10A	101 (2)	H30B—C30—H30A	98 (3)
C12—C11—C10	114.3 (4)	C32—C31—C30	112.8 (4)
C12—C11—H11A	91 (3)	C32—C31—H31B	100 (2)
C10—C11—H11A	111 (2)	C30—C31—H31B	113 (2)
C12—C11—H11B	101 (2)	C32—C31—H31A	116 (2)
C10—C11—H11B	108 (2)	C30—C31—H31A	107 (2)
H11A—C11—H11B	129 (3)	H31B—C31—H31A	109 (3)
C11—C12—H12A	109.5	C31—C32—H32A	109.5
C11—C12—H12B	109.5	C31—C32—H32B	109.5
H12A—C12—H12B	109.5	H32A—C32—H32B	109.5
C11—C12—H12C	109.5	C31—C32—H32C	109.5
H12A—C12—H12C	109.5	H32A—C32—H32C	109.5
H12B—C12—H12C	109.5	H32B—C32—H32C	109.5
			
C8—N1—C1—O1	179.0 (3)	C28—N2—C21—O3	178.2 (4)
C9—N1—C1—O1	0.6 (5)	C29—N2—C21—O3	5.0 (6)
C8—N1—C1—C2	−1.6 (3)	C28—N2—C21—C22	−0.5 (4)
C9—N1—C1—C2	180.0 (3)	C29—N2—C21—C22	−173.6 (3)
O1—C1—C2—O2	0.6 (5)	O3—C21—C22—O4	1.8 (6)
N1—C1—C2—O2	−178.8 (3)	N2—C21—C22—O4	−179.6 (3)
O1—C1—C2—C3	−179.3 (3)	O3—C21—C22—C23	−178.2 (3)
N1—C1—C2—C3	1.3 (3)	N2—C21—C22—C23	0.4 (3)
O2—C2—C3—C4	0.2 (6)	O4—C22—C23—C24	−2.7 (7)
C1—C2—C3—C4	−180.0 (3)	C21—C22—C23—C24	177.3 (4)
O2—C2—C3—C8	179.6 (3)	O4—C22—C23—C28	179.8 (4)
C1—C2—C3—C8	−0.5 (3)	C21—C22—C23—C28	−0.2 (4)
C8—C3—C4—C5	0.7 (5)	C28—C23—C24—C25	−0.3 (5)
C2—C3—C4—C5	180.0 (3)	C22—C23—C24—C25	−177.6 (4)
C3—C4—C5—C6	−0.5 (5)	C23—C24—C25—C26	−0.8 (5)
C4—C5—C6—C7	−0.2 (5)	C24—C25—C26—C27	0.8 (6)
C4—C5—C6—Br1	−179.9 (2)	C24—C25—C26—Br2	−179.0 (3)
C5—C6—C7—C8	0.8 (5)	C25—C26—C27—C28	0.2 (5)
Br1—C6—C7—C8	−179.6 (2)	Br2—C26—C27—C28	−180.0 (2)
C6—C7—C8—C3	−0.6 (4)	C26—C27—C28—C23	−1.3 (5)
C6—C7—C8—N1	−179.6 (3)	C26—C27—C28—N2	177.9 (3)
C4—C3—C8—C7	−0.1 (5)	C24—C23—C28—C27	1.4 (5)
C2—C3—C8—C7	−179.6 (3)	C22—C23—C28—C27	179.3 (3)
C4—C3—C8—N1	179.1 (3)	C24—C23—C28—N2	−177.9 (3)
C2—C3—C8—N1	−0.4 (3)	C22—C23—C28—N2	0.0 (4)
C1—N1—C8—C7	−179.6 (3)	C21—N2—C28—C27	−179.0 (3)
C9—N1—C8—C7	−1.2 (5)	C29—N2—C28—C27	−5.9 (5)
C1—N1—C8—C3	1.3 (3)	C21—N2—C28—C23	0.3 (4)
C9—N1—C8—C3	179.7 (3)	C29—N2—C28—C23	173.4 (3)
C1—N1—C9—C10	89.8 (4)	C21—N2—C29—C30	−106.6 (4)
C8—N1—C9—C10	−88.3 (4)	C28—N2—C29—C30	81.2 (4)
N1—C9—C10—C11	179.3 (3)	N2—C29—C30—C31	−177.1 (3)
C9—C10—C11—C12	172.4 (4)	C29—C30—C31—C32	174.8 (4)

### Hydrogen-bond geometry (Å, °)

**Table:**

*D*—H···*A*	*D*—H	H···*A*	*D*···*A*	*D*—H···*A*
C7—H7···O3^i^	0.95 (3)	2.44 (3)	3.367 (5)	165 (2)

Symmetry codes: (i) −*x*, −*y*, −*z*.
